# Alpha-Synuclein and GM1 Ganglioside Co-Localize in Neuronal Cytosol Leading to Inverse Interaction—Relevance to Parkinson’s Disease

**DOI:** 10.3390/ijms25063323

**Published:** 2024-03-15

**Authors:** Ranjeet Kumar, Suman Chowdhury, Robert Ledeen

**Affiliations:** Department of Pharmacology Physiology & Neuroscience, Rutgers, The State University of New Jersey, Newark, NJ 07103, USA; ranjeet.kumar1@rutgers.edu (R.K.); suman.chowdhury@rutgers.edu (S.C.)

**Keywords:** GM1, α-synuclein, NG108-15, Parkinson’s disease, neurodegeneration

## Abstract

Research on GM1 ganglioside and its neuroprotective role in Parkinson’s disease (PD), particularly in mitigating the aggregation of α-Synuclein (aSyn), is well established across various model organisms. This essential molecule, GM1, is intimately linked to preventing aSyn aggregation, and its deficiency is believed to play a key role in the initiation of PD. In our current study, we attempted to shed light on the cytosolic interactions between GM1 and aSyn based on previous reports demonstrating gangliosides and monomeric aSyn to be present in neuronal cytosol. Native-PAGE and Western blot analysis of neuronal cytosol from mouse brains demonstrated the presence of both GM1 and monomeric aSyn in the neuronal cytosol of normal mouse brain. To demonstrate that an adequate level of GM1 prevents the aggregation of aSyn, we used NG108-15 and SH-SY5Y cells with and without treatment of 1-phenyl-2-palmitoyl-3-morpholino-1-propanol (PPMP), which inhibits the synthesis/expression of GM1. Cells treated with PPMP to reduce GM1 expression showed a significant increase in the formation of aggregated aSyn compared to untreated cells. We thus demonstrated that sufficient GM1 prevents the aggregation of aSyn. For this to occur, aSyn and GM1 must show proximity within the neuron. The present study provides evidence for such co-localization in neuronal cytosol, which also facilitates the inverse interaction revealed in studies with the two cell types above. This adds to the explanation of how GM1 prevents the aggregation of aSyn and onset of Parkinson’s disease.

## 1. Introduction

Parkinson’s disease (PD) is the most prevalent of the synucleinopathies, a key feature of which is the accumulation of aggregated alpha-synuclein (aSyn) in the neurons of the central and peripheral nervous systems (CNS, PNS) [1]. That such aggregates, due to misfolded aSyn, are a key feature of PD pathology was fully illustrated in the landmark studies of Braak and others, featured in Braak’s staging hypothesis [2,3,4,5]. Those studies demonstrated early involvement of the olfactory and autonomic components of the CNS and PNS, which often occur many years prior to observable movement disorders in both genetic and sporadic forms of PD. These and later manifestations of PD pathology derive from the progressive loss of dopaminergic (DA) neurons, along with other neuronal types, due at least in part to the accumulation of these misfolded, aggregated aSyn formations known to occur in PD patients as Lewy bodies and Lewy neurites [6,7,8]. Our work [9,10] and that of others [8,11] showed the systemic deficiency of GM1 ganglioside in the brain and periphery and proposed it as the underlying cause of such aSyn aggregation; this was based at least in part on the application of GM1 to various animal models of PD with striking therapeutic results [12,13]. The present study was undertaken to provide more direct evidence for this critical effect of GM1 from studies of neural cells and neuronal cytosol.

Gangliosides are found in virtually all vertebrate cells but most prominently in neurons, where they have been extensively studied [14,15,16,17]. Ganglioside GM1 is one of these neuronal gangliosides and is therefore a major ganglioside in neuronal cells. It plays a vital role in several neuronal physiological processes such as cell signaling [18], memory [19], differentiation [20], neuroprotection [21], and apoptosis prevention [22]. Gangliosides also contribute to membrane organization and modulation of protein functions [23]. As organisms age, the metabolic functions of gangliosides, especially of the a-series (GM1, GD1a), progressively decline due to age-related progressive reduction in a-series ganglioside content [24,25], resulting in gradual neurological dysfunctions. GM1, with the assistance of GD1a (metabolic precursor to GM1), supports essential neuronal functions by interacting with proteins, promoting protein stereospecificity crucial for normal protein functioning [8,15,21,26,27]. Proteins such as brain-derived neurotrophic factor (BDNF) and nerve growth factor (NGF) have receptors that strongly bind with GM1, facilitating the functioning and long-term viability of neurons [28,29]. 

Alpha-synuclein is composed of 140 amino acids and is encoded by the *SNCA* gene. It has been well studied for at least three decades and has been shown to occur in the plasma membrane (in particular, synaptic membranes), cytosol, mitochondria, and nucleus [30]. While its normal neuronal functions are still under investigation, studies have reported its involvement in synaptic vesicle dynamics, mitochondrial function, intracellular trafficking, and chaperon activity [31,32]. Alpha-synuclein also exhibits neurotoxic properties when in oligomeric form, leading to progressive aggregation into proto-fibrils, more advanced fibrils, and finally Lewy bodies and Lewy neurites, as seen in PD patients [33]. It acts in the nucleus to promote neurotoxicity via the inhibition of histone acetylation [34]. The accumulation of aSyn increases with normal aging and a decline in proteolytic activity [35,36]. The ubiquitin–proteasome and lysosomal autophagy systems maintain intracellular homeostasis of aSyn, and any disruption in these mechanisms results in aSyn accumulation [36]. Aging, a well-established risk factor for PD, is closely associated with progressive impairment in ubiquitin–proteasome and lysosomal autophagy function [35]. The level of aSyn in the CNS and PNS is influenced by the balance between its synthesis, aggregation, and clearance. An imbalance in this system favors the accumulation, oligomerization and aggregation of aSyn, leading to PD-like symptoms [37]. Importantly, GM1 has been shown to promote the autophagy-dependent removal of aSyn [38].

Although the role of aSyn in neurons is not well established, studies have reported its interaction with negatively charged membrane lipids [39], suggesting its involvement in the fusion of synaptic vesicles with membranes [40]. Cytosolic aSyn occurs natively unfolded until it interacts with membranes, thereby acquiring an aggregated structure [41]. Several reports have shown the association of aSyn with lipids in PD [6,42,43,44]. GM1 interacts with α-Syn, inhibiting fibrillation, as observed in in vitro experiments where GM1 induced an α-helical structure, thereby interfering with aSyn fibril formation [45]. Notably, N-alpha-acetylation of aSyn increases its helical folding propensity and, thus, its binding specificity to GM1 and resistance to aggregation [46]. Thus, the aggregation of aSyn is a well-established hallmark of PD neuropathology, reported uniformly in virtually all PD patients.

Various investigations have reported in in vitro studies that physical as well as chemical factors like pH, ionic strength, temperature, and metal ions can affect aSyn aggregation to different extents [47,48], and aSyn has the maximum aggregating tendency with respect to other members (beta synuclein and gamma synuclein) of synuclein protein family [49]. Soluble and natively unfolded aSyn can self-assemble into amyloid fibrils [50] when incubated for a longer duration, which very closely resembles the fibrils obtained from PD patients’ brains [51].

To date, we have not found any reports describing the co-localization of aSyn and GM1. This is essential; if, for example, GM1 were entirely confined to the membrane (as has been asserted by some), there would be little opportunity for interaction with unfolded, soluble aSyn. Prior studies [see below] demonstrated a small but measurable portion of GM1 to be present as soluble component. The present study fortifies that with evidence showing the co-localization of GM1 and aSyn in neuronal cytosol owing to the co-release of these two neuronal components through mild homogenization of mouse brain. Prior cellular studies further pointed to their co-occurrence in a manner facilitating the inverse interaction now recognized as characteristic of these two neuronal components. These findings further illustrate the key role of GM1 in preventing aSyn aggregation with onset of PD.

## 2. Results

### 2.1. Alpha-Synuclein Aggregation and GM1 in Cultured Cells

**Figure 1:**
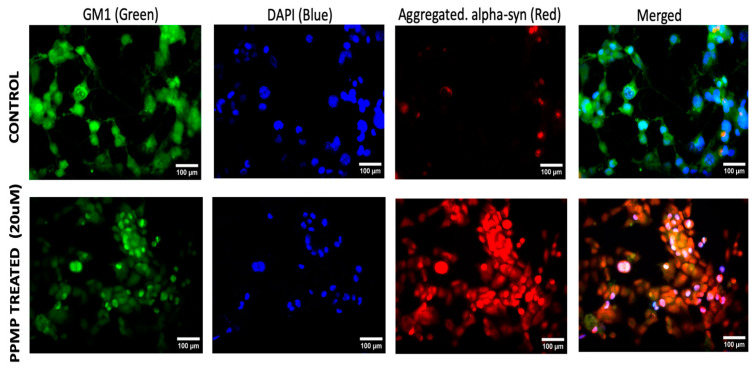
NG108-15 cells treated with PPMP: GM1 detected with CtxB linked to FITC (green) showed a marked decline. Aggregated α-Synuclein detected with antibody specific for that form of aSyn (red) showed marked elevation in misfolded aSyn (see Figure 2 for quantification).

**Figure 2:**
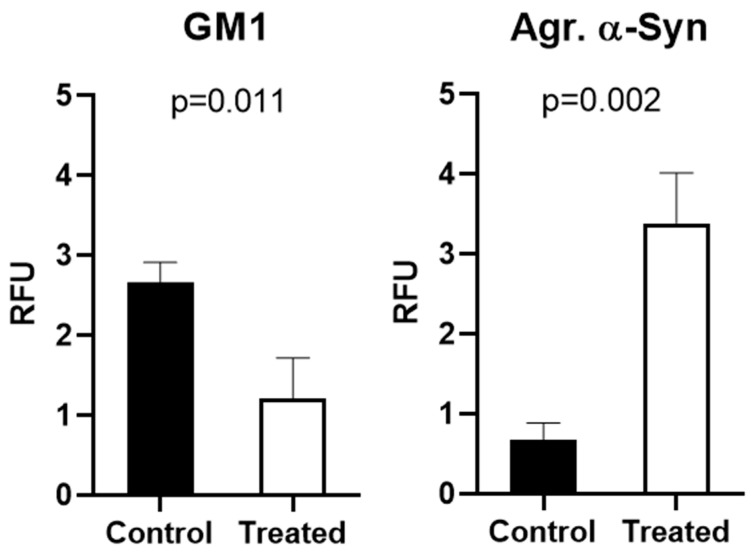
Quantification of above immunofluorescence images via ImageJ software. GM1 fluorescence intensity in PPMP-treated NG108-15 cells was greatly reduced, while aggregated aSyn was significantly increased compared to controls. Error bars indicate standard error of the mean.

The primary aim of this study was to demonstrate the co-localization of aSyn and GM1 within the neurons. We first employed NG108-15 cells, a neuroblastoma–glioma hybrid cell line widely used for in vitro studies in place of primary cultured neurons and shown to be effective as a model for studying neuronal function. We also used SH-SY5Y cells, a thrice-cloned cell line derived from the SK-N-SH neuroblastoma cell line which has been widely used for neurodegenerative studies [52,53,54]. For these cells in culture, we administered 20 μM PPMP, a structural analog of ceramide shown to inhibit UDP-glucose ceramide glucosyltransferase with a resultant decrease in glucosphingolipids such as GM1 [55]. Such treatment proved effective at inhibiting GM1 synthesis in these cells, compared to untreated control cells (Figure 1). Following staining with fluorescence-tagged antibodies specific to each molecule, we examined the cells with fluorescence microscopy, which revealed that the PPMP-treated cells exhibited reduced GM1 levels (green) along with an increase in aggregated aSyn (red). These changes were statistically significant (Figure 2). In contrast, the untreated cells displayed a significantly lower expression of aggregated aSyn and higher expression for GM1. Blue colored fluorescence represents nuclei which were stained with DAPI. The increase in aggregated aSyn in PPMP-treated NG108-15 cells is attributed to a decrease in endogenous GM1, required to bind to aSyn in order to retain it in a monomeric, non-aggregated state.

**Figure 3:**
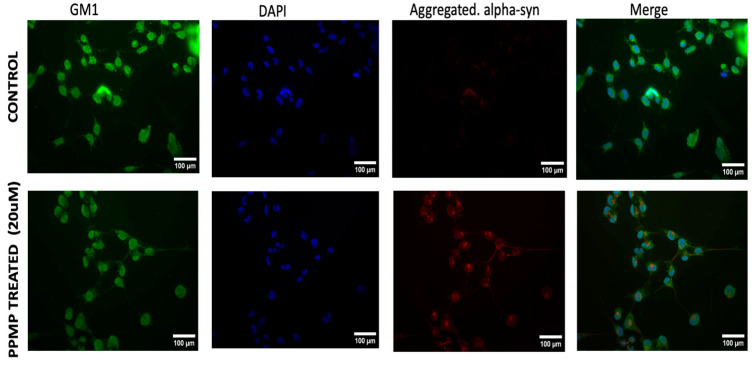
SH-SY5Y cells treated with PPMP: GM1 detected with Ctx-B linked to FITC showed marked decline. Aggregated α-Synuclein detected with antibody specific for aggregated aSyn showed marked elevation in aggregated aSyn (see Figure 4 for quantification).

**Figure 4:**
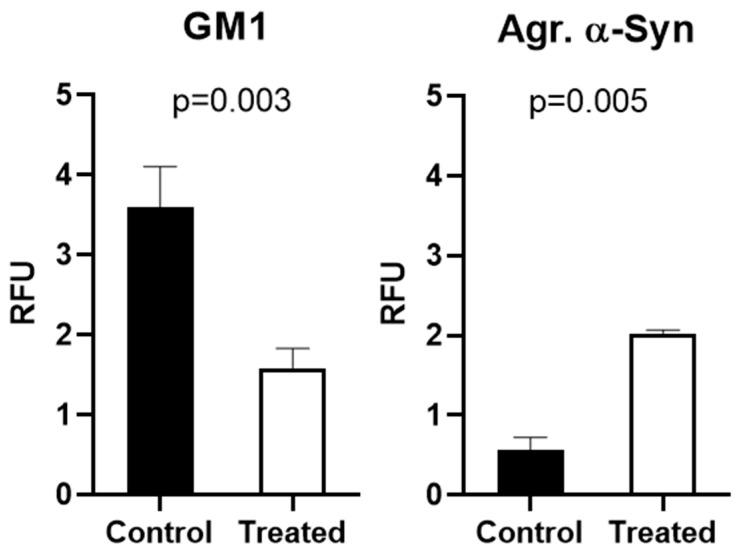
Quantification of immunofluorescence images of SH-SY5Y cells via ImageJ software. GM1 in treated cells was greatly reduced, while aggregated aSyn was significantly increased compared to controls. Error bars indicate standard error of the mean.

Figure 3 and Figure 4 show similar results for SH-SY5Y cells, with treatment with PPMP again causing a substantial decrease in GM1 (green) along with a significant elevation in aggregated aSyn (red), thus demonstrating again the inverse relation between aSyn and GM1. These results were statistically significant (Figure 4). This result thus applied to both cell types and we propose this as the operative mechanism for the remediation of PD symptoms in animal models of PD following the application of GM1—a form of GM1 replacement therapy. Subnormal GM1 was demonstrated in the previously employed mouse model of PD [6,12], analogous to the GM1 deficiency revealed in tissues from PD patients [9], and we have accordingly proposed the systemic deficiency of a-series gangliosides (GM1 and GD1a) as the underlying cause of PD [6]. Importantly, GM1 staining in both cell types was seen to fill the entire cell, including cytosol (as opposed to only plasma membrane staining), thus affirming the argument in favor of soluble cytosol as the locus of GM1–aSyn interaction.

Immunofluorescence images for GM1 and aSyn were quantified via ImageJ software (version 1.54h, NIH, Bethesda, MD, USA) (Figure 4). We observed that PPMP treatment caused a significant reduction in GM1 and equally significant elevation in aggregated aSyn compared to control cells. As with NG108-15 cells, this revealed the inverse relation between GM1 and aggregated aSyn.

### 2.2. Alpha-Synuclein and GM1 in the Neuronal Cytosol

**Figure 5:**
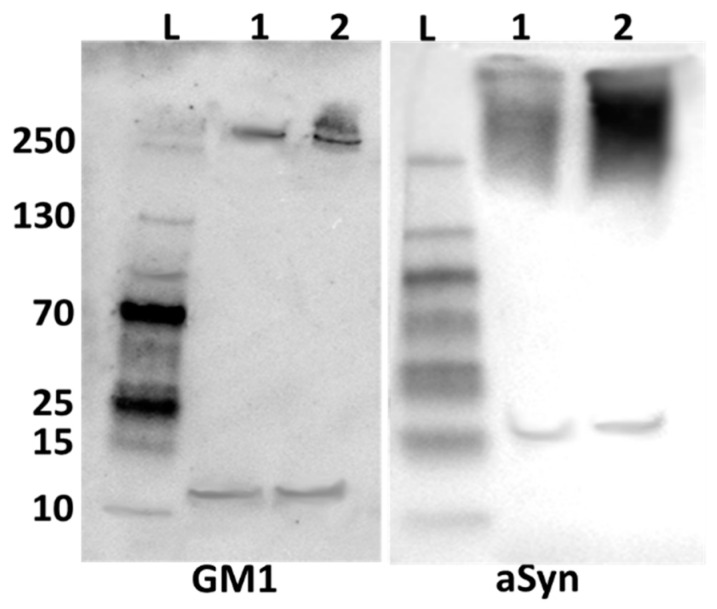
Alpha synuclein and GM1 both occur in neuronal cytosol and are released by both mild and vigorous homogenization (L: protein ladder, 1: manual homogenization, 2: notarized homogenization)Neuronal cytosol from mouse brain lysate was subjected to Native-PAGE: Electrophoresis at 50 volts for 3.5 h revealed the presence of both aggregated aSyn (~250 kDa) with associated GM1 and monomeric GM1 (11 kDa). Unassociated aSyn (16 kDa) was also present.

**Figure 6:**
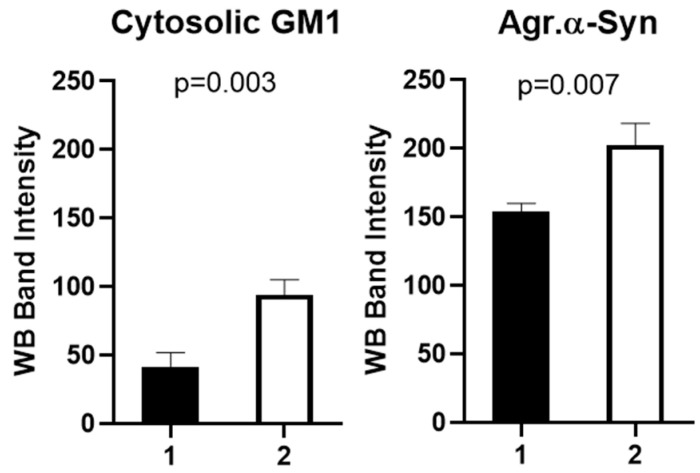
Quantification of changes in aggregated aSyn and associated GM1 resulting from mild vs. more vigorous homogenization of mouse brain (Figure 5). (1: manual homogenization, 2: notarized homogenization). Both changes were statistically significant. Error bars indicate standard error of the mean.

**Figure 7:**
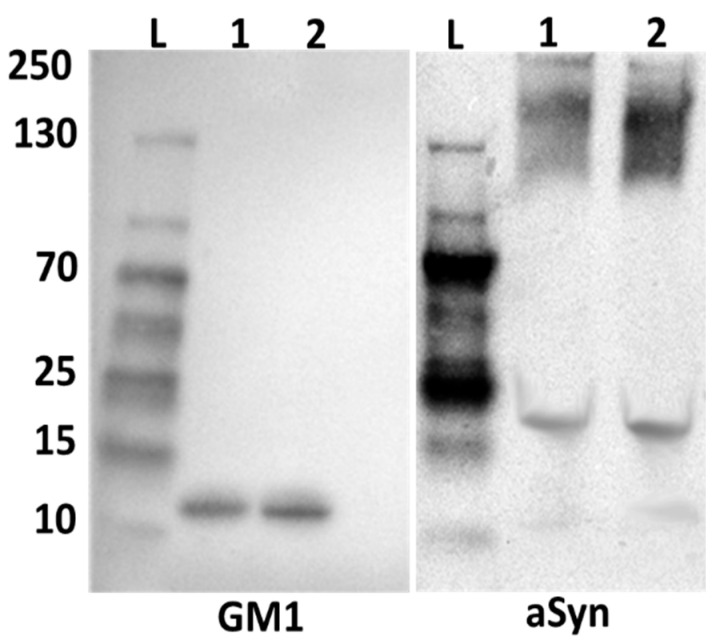
Alpha synuclein and GM1 are released from neuronal cytosol (see Figure 5) (L: protein ladder, 1: manual homogenization, 2: notarized homogenization). These mouse brain lysates were subjected to Native-PAGE. Electrophoresis at 55 volts (as opposed to the 50 V employed in Figure 5) for 3.5 h revealed only aggregated aSyn, showing more complete dissociation of GM1 from aSyn at the higher voltage (non-covalent linkage).

This study was designed to demonstrate the coexistence of GM1 and aSyn in neuronal cytosol, pointing to their non-covalent association at that locus; we propose that this interaction is what prevents the aggregation of aSyn. For this, we employed Western blot analysis applied to cytosolic lysates from mouse brain neurons, prepared by both mild and more vigorous homogenization. For mild homogenization, we employed a hand-held glass tube and Teflon pestle, all kept at 4 °C; more intensive homogenization was achieved with a motor-driven homogenizer. These produced aggregated aSyn in both cases, considerably less with mild homogenization (Figure 5, Figure 6). GM1 was bound to the aggregated aSyn but was also present in free form, as was also true for some non-aggregated aSyn. The aggregated aSyn undoubtedly resulted from the interaction of the monomeric form with membranes during homogenization and as it exited the neuron (see below and Discussion), while this aggregated form was still able to bind to GM1. The operative electrophoresis in that experiment was 50 V, sufficient to allow for some remaining GM1-aSyn association, in contrast to somewhat stronger electrophoresis (55 V), which resulted in more complete dissociation of GM1 from aSyn (Figure 7). This relatively facile dissociation pointed to a non-covalent linkage between the two molecules.

We also carried out an assessment of GM1 and aSyn joint occurrence in mouse neuronal cytosolic lysate by means of Native-PAGE electrophoresis and Western blot analysis. This revealed distinct variations in the aggregation of aSyn when homogenizing tissue with different conditions. In Figure 5, it is evident that samples subjected to more intensive homogenization exhibited greater levels of aggregated aSyn compared to gentler homogenization. This higher level of aggregated aSyn likely revealed the result of monomeric aSyn association with membranes (non-gangliosidic) during homogenization, as previously shown [41]. GM1 associated with this aSyn was similarly elevated as revealed with CtxB. In that result, the GM1 band appeared at 11 kDa rather than its true molecular weight (1548 D), as reported in another study [56]. Aggregated aSyn appeared at its expected molecular weight. Furthermore, our observations again indicated that higher levels of GM1 correlated with less aggregated aSyn, while lower levels of GM1 were associated with more aggregated aSyn.

The question might well be asked as to how we identified the lysates as neuronal in origin, rather than as astrocytic for example, a considerably more abundant cell type in the brain. The response to that is revealed in previous work showing that astrocytes do not produce their own GM1 ganglioside [57,58,59]. Concerning other types of brain cells, it seems unlikely the mild homogenization employed would produce similar lysates. Moreover, the fact that aSyn, a neuron-specific molecule, was released provides further evidence that the lysates were neuronal in origin.

## 3. Discussion

Gangliosides have been widely viewed for some years as exclusively membrane-localized components in neurons, but, in fact, they are present as intracellular components as well, including soluble cytosol [60,61]. These cytosolic, soluble gangliosides were shown to bind to soluble proteins, pointing to the presence of at least one GM1–protein complex, although the specific protein(s) to which GM1 bonded was not identified [60]. The present study presents evidence that at least one such GM1-binding protein in cytosol is aSyn; that association is believed to be what inhibits aSyn fibrillation, thus demonstrating the significant role of GM1 in averting Lewy body formations.

GM1 is a widely studied ganglioside, which, along with several other ganglio-series gangliosides (GD1a, GD1b, GT1b, etc.), is present most abundantly in neurons of the CNS and PNS [14]. Among the many proteins with which GM1 is functionally associated is aSyn, described as an almost entirely soluble, monomeric protein localized to the synapse and nucleus [30,31,60,62] with a synaptic function that is not entirely known (its function in the nucleus appears to be pathological). It is prone to aggregate under adverse (e.g., pathological) conditions to form proto-fibrils and pathological fibrils, leading to the formation of Lewy bodies and Lewy neurites. These are the well-recognized hallmarks of PD [63] that were used by Braak and coworkers in developing their six-stage hypothesis of PD [2,3]. This points to the question of what prevents such aggregation in the normal state, and our previous studies suggested aSyn association with GM1 holds this protein in its non-aggregating, helical conformation [64]. This was in accordance with studies showing that GM1 binds to aSyn with high affinity and specificity [45], an association enhanced by the N-terminal acetylation of aSyn [46]. This was proposed as the function of the small pool of soluble GM1 in neuronal cytosol [60,61] that was found to bind soluble proteins of unknown identity [60]. In view of the present findings, it seems likely that these soluble proteins include aSyn, and the growing decrease in GM1 in this soluble pool would account for the gradual increase in aSyn fibrillation and Lewy bodies’ formations.

Gangliosides are present in virtually all mammalian cells, most abundantly in neurons where they mediate numerous neuronal functions [15,64,65,66,67,68,69]. The key role of GM1 in several of these functions has been highlighted [7,21,26,64,70], along with its progressive decline with aging [24,25]. A similar age-related decrease in GM1 was recently demonstrated in peripheral tissues of normal mice with full expression of B4galnt1 [71]. An additional finding of Svennerholm et al. was significant variations in the level of a-series gangliosides (GM1, GD1a) among individuals of the same age [24,25]. These age-related decreases in GM1, when pronounced and often enhanced by additional distortions (e.g., defective lysosomal hydrolyses), have been associated with the onset of Parkinsonism. Thus, the systemic deficiency of GM1 has been demonstrated in several tissues of PD patients [9], even including peripheral blood mononuclear cells [10], a cell type not intimately associated with neurons. Accordingly, the key role of GM1 deficiency in PD has been recently highlighted along with the physiological consequences of such a deficiency [7,26,64,70].

The predominant role of GM1 deficiency became evident in work with B4galnt1-null mice that possess total ganglio-series ganglioside deficiency (including GM1), resulting in impaired movement and various neuropathological symptoms of PD, e.g., elevation and aggregation of aSyn, loss of tyrosine hydroxylase (TH)-positive dopamine (DA) neurons in the substantia nigra pars compacta (SNpc), depletion of striatal DA, and reduced pRet expression in TH+ neurons [72]. Of special interest was the observation that heterozygous mice (B4galnt1+/−), with only partial GM1 deficiency showed similar PD symptoms [71], similar in magnitude to the deficiencies observed in PD patients, e.g., occipital cortex, nigrostriatal neurons of the SNpc [12], and extra-CNS sites representing the PNS [9]. These findings point to the expression of body-wide symptoms that similarly characterize PD.

The question arises as to why the possibility of astrocytic ganglioside was not considered. Previous work showed that astrocytes do not produce their own GM1 gangliosides, with neurons being the only major source of this type of ganglioside [57,58]. Thus, this more abundant cell type was not the likely source of the GM1 released by mild homogenization of brain tissue. The same would apply to the other cell types of brain.

It was noteworthy that the aSyn fibrillation that accompanied such GM1 deficiency in mouse models of PD could be dispersed by peripheral application of GM1 or its oligosaccharide, as well as by LIGA20 (analog of GM1) [12,73]. In addition, such aggregates were resolved by GM1 treatment in rats with the aSyn model of PD [13]. However, we considered it essential to further elucidate this GM1/aSyn interaction, as now provided in the present study. Western blot analysis of GM1 and aSyn revealed the association of these entities (aggregated aSyn) after release from neuronal cytosol by both mild and stronger homogenization followed by relatively mild electrophoresis (50 volts) (Figure 5 and Figure 6). Moreover, electrophoresis at higher voltage (55 volts) resulted in the dissociation of GM1 from aggregated aSyn, while both unassociated GM1 and monomeric aSyn were still detectable (Figure 7). Some research groups have reported in vitro investigation into the interaction between GM1 and aSyn [41]. However, a literature review failed to uncover any studies demonstrating the cytosolic (in vivo) association between GM1 and aSyn.

Several studies have reported the oligomerization and aggregation of aSyn under the influence of different physical and chemical factors. In view of this, we used two different homogenization procedures before extracting cytosolic lysate from the mouse brains. One half of each mouse brain was homogenized manually very gently on ice, while the other half was homogenized more vigorously with a motorized homogenizer in short pulses on ice. The cell lysates were subjected to electrophoresis and we obtained a differential aggregation of aSyn in both cell lysates. Manually homogenized lysate showed less aggregation of aSyn, whereas motorized homogenization imparted more aggregation of aSyn. This difference was consistent with the likely difference in association of aSyn with cellular membranes [41], a factor to be taken into account when considering aSyn association with GM1.

By using Native-PAGE Western blot at varying voltages, we observed an association between GM1 and aSyn in the neuronal cytosol of the mouse brain. Intriguingly, when subjected to electrophoresis at 55 volts, a more complete dissociation of GM1 from aSyn occurred than we observed at 50-volt electrophoresis, indicating a weak non-covalent interaction vulnerable to mild stress, such as electrostatic forces. At 50 volts, GM1 was observed to localize with aggregated aSyn. This slight increase in electrophoresis voltage caused more complete GM1 dissociation from aSyn at 55 volts.

Out of the 140 amino acids that comprise the structure of aSyn, a hydrophobic stretch of 12 amino acids (71-VTGVTAVAQKTV-82) makes it prone to aggregation [49]. Any addition or deletion of a charged residue in this hydrophobic stretch dramatically hampers the amyloid formation capacity of aSyn, which signifies the importance of this hydrophobic stretch in aSyn aggregation [74]. It is not known if this is the binding site for GM1, although this seems likely in view of the dramatic effect of GM1 on aSyn aggregation. In any case, it is clear that a primary requirement for GM1 interaction with aSyn is co-localization of these two substances within the neuron, which this study has attempted to provide. In addition, the application of 20 uM PPMP to both cells resulted in a significant decrease in GM1 together with a marked elevation in aggregated aSyn (Figure 1 and Figure 3), further emphasizing the inverse relationship between these two substances. These results add to the growing evidence that GM1 is able, in part through the interaction with aSyn, to block and even reverse that aspect of neuronal decline, leading to PD.

## 4. Materials and Methods

### 4.1. Materials

Cholera Toxin B subunit-(FITC) conjugate (Cat# C1655, Sigma, St. Louis, MO, USA), rabbit monoclonal anti-alpha-synuclein aggregate (Cat# ab209538, Abcam, Trumpington, Cambridge, UK) and goat anti-rabbit IgG (H+L) secondary antibody, Texas Red-X (Cat#T6391, Invitrogen, Carlsbad, CA, USA) were purchased from Sigma, Abcam and Invitrogen, respectively. D-*threo*-1-phenyl-2-Palmitoylamino-3-morpholino-1-propanol (PPMP) was from Cayman Chemical (Cat# 22677, Ann Arbor, MI, USA) and 4′,6-Diamidino-2-Phenylindole, Dihydrochloride (DAPI) was purchased from Cat#D1306, Invitrogen, Carlsbad, CA, USA, poly-L-Lysine (Cat# A3890401, Gibco, Waltham, MA, USA), anti-fade sealing agent (Cat# ab104135, Waltham, MA, USA). All other chemicals used were from Sigma, St. Louis, MO, USA.

Sucrose (Sigma, St. Louis, MO, USA), protease inhibitor (Halt, Thermo, Waltham, MA, USA), Pierce BCA Protein assay kit (Thermo Scientific, Waltham, MA, USA), Mini-PROTEAN TGX Precast Gel (Cat# 4561094, Bio-Rad, Hercules, CA, USA), Tris-buffered saline with Tween 20 (TBST) (Cat# 28360, Thermo Scientific, Waltham, MA, USA), PVDF membrane (Trans-Blot Turbo Transfer, Cat# 1704156, Bio-Rad, Hercules, CA, USA), Cholera Toxin subunit B-HRP conjugate (Cat# c34780, Sigma, St. Louis, MO, USA), mouse mab aSyn antibody (Cat # 2A7, Novus, Centennial, CO, USA) and goat anti-mouse HRP conjugated secondary antibody (Cat# a28177, Therma fisher, Waltham, MA, USA) were purchased for Western blot.

### 4.2. NG108-15 and SH-SY5Y Cellular Studies

For each of these cells, we studied the immunofluorescence of neuronal cytosolic GM1 interaction with aSyn. Briefly, both cells were maintained up to 90% confluency followed by counting live cell density. Sterilized coverslips coated with poly-L-Lysine 0.1 mg/mL, were prepared one day in advance. The cells (50,000 cells/well) were plated on coverslips and incubated in CO_2_ incubator for 3 h at 37 °C. This was followed by treatment of the cells with PPMP (20 µM) and further incubation for 48 h at 37 °C. The cells were then fixed for 15 min at room temperature in 2% paraformaldehyde (in PBS at pH 7.4), followed by washing with 1X PBS for 5 min. The cells were then permeabilized and simultaneously blocked for 60 min in PBS + 0.1% Triton X-100 + 10% FBS solution. Primary antibodies were diluted in PBS + 0.1% Triton X-100 + 10% FBS solution (Cholera Toxin B subunit-(FITC) conjugate, 1.5 µg/mL; anti-alpha-synuclein aggregate, 0.2 µg/mL) and incubated together with permeabilized cells overnight at 4 °C. Next day, antibody solution was removed, and the cells were washed with 1X PBS twice for 5 min. For the binding of secondary antibodies conjugated with Texas red (Goat anti-Rabbit IgG (H+L) Texas Red-X, 5 µg/mL), the cells were incubated for 2 h at room temperature before being washed twice for 5 min with 1X PBS. The cells were further incubated with DAPI (0.5 µg/mL) for nuclear staining for 15 min at room temperature, followed by two washing with 1X PBS for 5 min. For mounting coverslips on slides, fluoroshield medium was used, and images were acquired on an Olympus BX51 microscope, using a 40× objective. A comparison was made between GM1 staining of whole cells and that of cell plasma membranes.

### 4.3. Western Blot Analysis

The mice were maintained at the animal facility of the Rutgers New Jersey Medical School Animal Facility. This study was ethically approved by the Institutional Animal Care and Use Committee (IACUC, Rutgers University). B4galnt1 heterozygous (HT) and the wild type (WT) mice (C57BL/6J background) were maintained on a standard pellet diet ab libitum and bred in the animal facility of Rutgers New Jersey Medical School. Genotyping was carried out by Transnetyx Inc. (Cordova, TN, USA) using tail snips.

The cytosolic fraction was collected from normal mouse brains, as described by Sonnino et al. [60], with some modifications. The mice were euthanized, and the dissected brains were weighed, and one half was homogenized in five volumes of 0.25 M sucrose solution with 1 mM potassium phosphate buffer and protease inhibitor, phosphatase inhibitor (in 0.1 mM EDTA + 10 mM sodium fluoride, pH 7.2). A sterilized glass homogenizer with Teflon pestle at 4 °C was employed to homogenize the tissue manually for 2 min (gently); the other brain half was homogenized more vigorously with a motorized homogenizer in small pulses until the tissue was completely homogenized. Both samples were then centrifuged at 150,000× *g* for 1 h at 4 °C. The supernatants were carefully collected; each pellet was again washed with half of the initial volume of 0.25 M sucrose solution and centrifuged again for one hour at 150,000× *g*. After centrifugation, the supernatant was carefully collected and mixed with the previously collected supernatant. Protein was then estimated with bicinchoninic acid (BCA) assay, as per the manufacturer’s protocol. Equal amounts of 25 µg of cytosolic protein were loaded on pre-casted gels (Bio-Rad, Hercules, CA, USA) at 4 °C and separated on Native-PAGE, 4–20% Mini-PROTEAN TGX, 50 V and 55 V for 3.5 h. Resolved proteins were transferred to PVDF membrane with 50 V for 30 min at 4 °C. Blot was immersed in blocking buffer (5% skimmed milk in TBST) for one hour at room temperature, whereas for aSyn, before blocking, blot was immersed in 0.4% paraformaldehyde for 30 min, followed by washing 3× with nuclease-free water for 5 min. Blocked PVDF membranes were incubated overnight with 1:1000 dilutions of aSyn antibody and cholera toxin subunit B (HRP conjugated). Proteins were visualized after incubation with HRP-conjugated second antibody (for aSyn) with chemiluminescence (ECL, Life Technologies, Frederick, MD, USA) as per the manufacturer’s instructions. Developed blots were viewed and analyzed with chemi-blot imager (Invitrogen, Carlsbad, CA, USA).

### 4.4. Image Quantification and Statistical Analysis

All images were quantified with ImageJ software (version 1.54h, National Institute of Health (NIH), Bethesda, MD, USA; website: https://imagej.nih.gov/ij/), and graphs were prepared with Graph Pad Prism 8. Statistical analysis was determined via Student’s *t*-test.

## 5. Conclusions

GM1 and aSyn occur together in neuronal cytosol. A decrease in GM1 in NG108-15 and SH-SY5Y cells (via PPMP treatment) resulted in a marked increase in aggregated aSyn, emphasizing the inverse relation of these two molecules. Western blot analysis revealed the formation of both unassociated GM1 and aSyn as separate entities. In addition, GM1 was partially associated with aggregated aSyn at a lower voltage (50 V); a higher voltage (55 V) caused the release of all GM1 from aggregated aSyn, indicating non-covalent linkage. These results substantiate the associations of soluble, linear aSyn with GM1 in the small pool of soluble cytosolic GM1 as the mechanism that prevents the aggregation of aSyn in normal neurons.

## Data Availability

Data are contained within the article.
